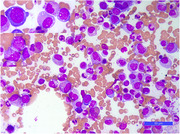# Vexas: A new syndrome not always associated with hemopathy

**DOI:** 10.1002/jha2.341

**Published:** 2021-12-01

**Authors:** Benoît Thomas, Julien Campagne

**Affiliations:** ^1^ Laboratoire de Biologie Médicale UNEOS Metz France; ^2^ Médecine interne UNEOS Metz France

A 63‐year‐old male presented with polyarthralgia and a purpuric rash on the lower extremities associated with fatigue and weight loss. Clinically, we noticed a subcutaneous nodule on his left arm, and its biopsy revealed a septal panniculitis with dermo‐hypodermic vasculitis consistent with a polyarteritis nodosa. Biopsy of the petechial purpura was also performed, and it revealed leukocytoclastic vasculitis. A glucocorticoid regimen was started.

C‐reactive protein (CRP) levels decreased and the treatment was tapered. After 8 weeks, hand arthralgia and lower limb myalgia reappeared with a concomitant increase in CRP levels. Azathioprine was introduced. After an improvement phase, CRP levels rose again, and a regimen of prednisone and cyclophosphamide was initiated. Their clinical condition improved, without any pain or cutaneous manifestations, but CRP levels remained high. Evaluation by means of a bone marrow aspiration was performed. No myeloma and no dysplasia were found, but myeloid and erythroid precursors exhibited numerous cytoplasmic vacuolations. This observation justified a search for *UBA1* mutation. *UBA1* was found to be mutated, thus confirming the diagnosis of VEXAS. Anti‐interleukin‐6 receptor treatment (tocilizumab) was introduced. Contrary to our observation, VEXAS (vacuoles, E1 enzyme, X‐linked, autoinflammatory, somatic) syndrome, which is a newly described adult‐onset autoinflammatory disease, has been described as being associated with myelodysplastic syndrome or myeloma.